# Fermentative Synthesis of Gluconic and Xylonic Acids from Hydrolyzed Palm Fronds Using *Gluconobacter oxydans*

**DOI:** 10.3390/bioengineering12080801

**Published:** 2025-07-25

**Authors:** Ibnu Maulana Hidayatullah, Dhea Annora Maritza, Masafumi Yohda, Muhammad Sahlan, Adi Kusmayadi, Yoong Kit Leong, Heri Hermansyah

**Affiliations:** 1 Department of Chemical Engineering, Faculty of Engineering, Universitas Indonesia, Depok 16424, Indonesia; ibnu.maulana.h@che.ui.ac.id (I.M.H.); dheannoraa@gmail.com (D.A.M.); 2 Research Center for Biomedical Engineering, Faculty of Engineering, Universitas Indonesia, Depok 16424, Indonesia; 3 Research Center for Biomass Valorization, Universitas Indonesia, Depok 16424, Indonesia; 4 Division of Biotechnology and Life Science, Institute of Engineering, Tokyo University of Agriculture and Technology, Tokyo 183-8538, Japan; yohda@cc.tuat.ac.jp; 5 Department of Mechanical Engineering, Politeknik Negeri Indramayu, Indramayu 45252, Indonesia; adikusmayadi@polindra.ac.id; 6 Department of Chemical and Materials Engineering, Tunghai University, Taichung 407224, Taiwan; yoongkit1014@thu.edu.tw; 7 Research Center for Smart Sustainable Circular Economy, Tunghai University, Taichung 407224, Taiwan

**Keywords:** waste valorization, microbial fermentation, gluconic acid, xylonic acid, sustainable bioconversion

## Abstract

The escalating demand for sustainable and eco-friendly production processes has necessitated the exploration of renewable resources for the synthesis of valuable chemicals. This study investigated the fermentative synthesis of gluconic acid (GA) and xylonic acid (XA) from hydrolyzed palm fronds by using *Gluconobacter oxydans*. The key variables examined included agitation speed, inoculum ratio, and composition of fermentation media. In a synthetic medium, maximum GA concentration reached 52.82 ± 12.88 g/L at 65 h using 150 rpm agitation and 15% (*v*/*v*) inoculation, while maximum XA concentration achieved 2.31 ± 1.43 g/L at 96 h using 220 rpm agitation and 9% (*v*/*v*) inoculation. In the hydrolysate medium, the maximum GA concentration was 3.24 ± 0.66 g/L at fermentation onset using 220 rpm agitation and 15% (*v*/*v*) inoculation, while the maximum XA concentration reached 0.62 ± 0.04 g/L at 24 h using 190 rpm agitation and 5% (*v*/*v*) inoculation. These findings demonstrate the feasibility of utilizing palm fronds as a renewable feedstock for the sustainable synthesis of high-value biochemicals, promoting waste valorization, and contributing to the advancement of a circular bioeconomy.

## 1. Introduction

Oil palm is an essential industrial crop that is widely valued for its versatility in the production of industrial oils, cooking oils, and renewable biofuels. In Indonesia, oil palm cultivation has expanded dramatically over the past century, reaching 16.8 million hectares by 2023 [1]. While this industry supports economic growth and global supply chains, it also generates considerable biomass waste, including empty fruit bunches, fronds, fruit fibers, and shells. Among these by-products, palm fronds are particularly underutilized and commonly repurposed only as compost or animal feed, with much left to accumulate in plantations, posing an environmental challenge [2].

Palm fronds are distinctive in their high holocellulose content, comprising 80–83% cellulose, hemicellulose, and lignin, making them superior in fiber content compared to other palm parts, such as empty fruit bunches (68–86%), trunks (42–45%), and mesocarp (70–71%) [3] This composition makes palm fronds excellent candidates for chemical and biological processing. The high cellulose and hemicellulose contents provide a source of fermentable sugars, whereas the lignin content offers additional potential for bio-based applications [4]. The utilization of palm fronds in advanced processing methods can convert this biomass into valuable products, thereby contributing to sustainable waste management and value-added production.

Through hydrothermal pretreatment and enzymatic hydrolysis, palm-frond holocellulose can be converted into fermentable sugars, such as glucose and xylose [5]. These sugars serve as essential precursors for valuable organic acids used in various industries [6]. Gluconic acid (GA) and xylonic acid (XA) are notable organic acids that are derived from these compounds. GA, initially discovered in *Aspergillus niger* and now commonly produced by bacteria such as *Gluconobacter*, *Pseudomonas*, and *Acetobacter* [7], has widespread applications in the food, pharmaceutical, and cleaning industries. XA recognized by the U.S. The Department of Energy, one of the top 30 bio-based chemicals, is used in the pharmaceutical, food, and construction industries [8].

*Gluconobacter oxydans*, a Gram-negative member of the acetic acid bacteria family, is highly effective in producing GA and XA simultaneously through fermentation. This unique metabolic pathway enables the oxidant to oxidize glucose to GA and convert xylose to XA, making it a suitable biocatalyst for efficient and economical bioconversion [9,10]. Fermentation with *G. oxydans* is a cost-effective alternative to chemical synthesis, and it offers environmental and economic advantages.

Although prior studies have explored hydrolysates from biomass sources, such as corn and potato waste, as substrates for microbial fermentation [11,12], the use of palm fronds remains a novel approach. Given their high holocellulose content, palm fronds have promising potential as sustainable feedstocks for GA and XA production [3]. This study aimed to evaluate the potential for producing GA and XA using both synthetic medium and palm frond hydrolysate during fermentation with *G. oxydans*, to optimize process parameters, and to assess product yields to develop a sustainable bioconversion platform.

## 2. Materials and Methods

### 2.1. Materials and OPF Hydrolysate Preparation

Oil palm fronds (OPF) were sourced from PT Perkebunan Nusantara VIII, a state-owned agro-industrial enterprise located in Bogor, West Java, Indonesia. Dried OPF was milled to achieve a uniform particle size of 40–80 mesh. Hydrothermal pretreatment was conducted with a solid loading (SL) of 12% using distilled water as the solvent at 165 °C for 1 h in a 5 L hydrothermal reactor (Model SPC1-35-E, made of stainless steel 316, CV. Pugar Mandiri, Bandung, Indonesia) to disrupt the lignocellulosic structure and improve cellulose accessibility.

Following hydrothermal pretreatment, the pretreated OPF underwent enzymatic hydrolysis (99.5 FPU/g of commercial cellulase and 14,735 U/mL of commercial xylanase) with enzyme loading of 0.2% *w*/*v* and 0.05 M (pH 5) citric acid buffer addition in a shaking incubator at 50 °C, working volume of 200 mL; and 150 rpm for 72 h. The resulting OPF hydrolysate was centrifuged at 6000 rpm for 10 min and subsequently filtered through Whatman No.1 filter paper to remove any solid residues. The filtered OPF hydrolysate, containing 3.37 ± 0.09 g/L glucose and 2.12 ± 0 g/L xylose, was sterilized in an autoclave at 121 °C for 15 min [13].

Additional materials, including D(+)-Glucose, D(+)-xylose, yeast extract, and potassium dihydrogen phosphate, were obtained from Merck KGaA (Darmstadt, Germany). Ammonium sulfate was obtained from Smart-lab (Tangerang, Indonesia), and magnesium sulfate heptahydrate and tryptone were supplied by the Central Drug House (CDH) in New Delhi, India. Standards for HPLC, specifically D-Gluconic acid and D-Xylonic acid lithium salt, were procured from Sigma-Aldrich (St. Louis, MO, USA).

### 2.2. Strain and Media

*G. oxydans* DSM 46616 from the German Collection of Microorganisms and Cell Cultures (Braunschweig, Germany), was cultured in 50 g/L glucose, 5 g/L tryptone, 20 g/L yeast extract, 0.5 g/L MgSO_4_·7H_2_O, 1.5 g/L KH_2_PO_4_, and 1.5 g/L (NH_4_)_2_SO_4_; and a synthetic fermentation medium (as a control variable) was prepared with the following composition of 50 g/L glucose, 25 g/L xylose, 5 g/L tryptone, 20 g/L yeast extract, 0.5 g/L MgSO_4_·7H_2_O, 1.5 g/L KH_2_PO_4_, and 1.5 g/L (NH_4_)_2_SO_4_.

The inoculum was prepared in a 500 mL Erlenmeyer flask containing 300 mL of the seed medium. The culture was incubated at 30 °C with continuous shaking at 220 rpm for 72 h, achieving an OD_600_ value between 1 and 2, indicating that the cells were ready for inoculation into the fermentation medium [14].

### 2.3. Gluconic Acid and Xylonic Acid Fermentation

Fermentation experiments were conducted using synthetic and OPF hydrolysate media in a 500 mL Erlenmeyer flask with a working volume of 300 mL. To evaluate the optimal growth conditions and microbial performance, inoculum ratios of 5% (*v*/*v*), 9% (*v*/*v*), and 15% (*v*/*v*) were tested at agitation speeds of 150 rpm, 190 rpm, and 220 rpm. Fermentation was maintained at a constant temperature of 30 °C over a 96 h period in an incubator shaker, ensuring aerobic conditions throughout the process to promote efficient microbial activity and metabolite production [14].

### 2.4. Analytical Methods

Cell growth was analyzed using gravimetric and turbidimetric methods. For gravimetric analysis, the dry weight of the cells was determined by harvesting the cell pellets, washing them to remove impurities, and drying them at 105 °C for 1 h in an oven before weighing. The turbidity method involved measuring the optical density (OD_600_) of the culture using a UV-VIS spectrophotometer (UV-1280, Shimadzu, Kyoto, Japan). OD_600_ values were calibrated against cell dry weight using a standard curve, enabling a comprehensive assessment of biomass production and microbial activity during fermentation [13,15].

GA and xylonic XA concentrations were analyzed using an HPLC (UV/VIS detector, Shimadzu, Kyoto, Japan) equipped with an Aminex HPX-87H column (Bio-Rad, Hercules, CA, USA) at 55 °C with 5 mM H_2_SO_4_ as the mobile phase at a flow rate of 0.4 mL/min. Glucose and xylose levels were measured as substrates using HPLC (RI detector, Shimadzu, Kyoto, Japan) with the same Aminex HPX-87H column at 60 °C, using 5 mM H_2_SO_4_ as the mobile phase at a flow rate of 0.6 mL/min [9].

### 2.5. Statistical Analysis

The experiments were conducted in triplicate, and the resulting data were statistically analyzed using Microsoft Excel 365. One-way analysis of variance (ANOVA) was performed using MiniTab 17.1.0, with the significance level set at *p* < 0.05, to determine the differences between treatments.

## 3. Results and Discussions

### 3.1. Effect of Inoculum and Speed Agitation on Gluconobacter oxydans Growth

**Figure 1:**
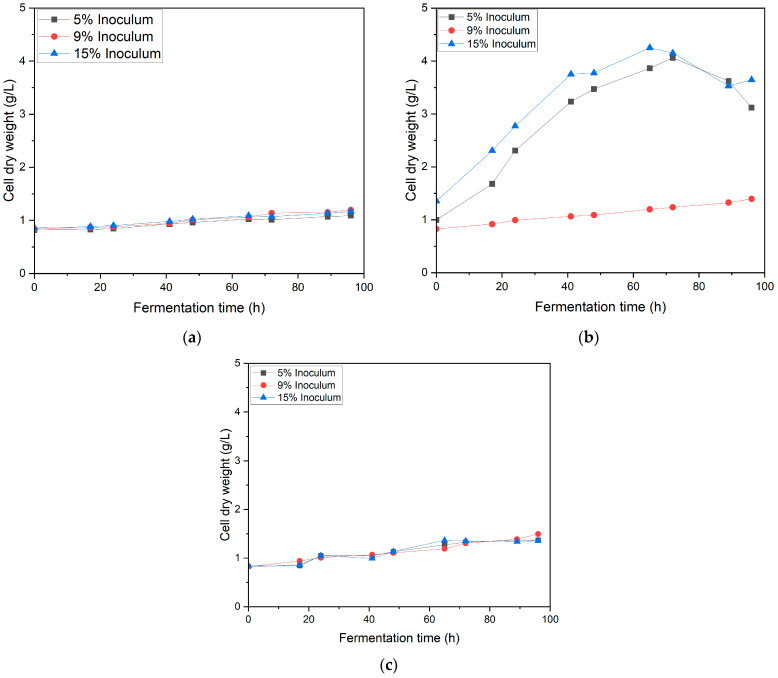
*G. oxydans* growth profile in various inoculum ratios (5%, 9%, and 15%) at agitation speeds (**a**) 150 rpm, (**b**) 190 rpm, (**c**) 220 rpm.

Figure 1 shows the impact of inoculum size (5%, 9%, and 15%) and agitation speed on the growth of *G. oxydans*. Under agitation at 150 rpm (Figure 1a), all inoculum sizes exhibited similar growth trends, reaching approximately 0.9–1.1 g/L at 96 h, indicating the limited influence of inoculum size under this condition. High agitation (Figure 1b) significantly enhances growth for the 15% inoculum, which peaks at around 4 ± 1.40 g/L at 60–70 h, while the 5% inoculum reaches 3.5 ± 1.30 g/L and the 9% inoculum plateaus at about 1 ± 0.1 g/L, suggesting that higher agitation improves oxygen transfer but may lead to suboptimal conditions for certain inoculum sizes. Medium agitation (Figure 1c) shows comparable trends to low agitation, with all inoculum sizes achieving about 1.2–1.4 g/L at 100 h, indicating sufficient oxygenation without drastic impacts on growth [16,17]. Overall, 15% inoculum combined with high agitation was the most favorable condition, maximizing growth due to improved oxygen availability and substrate utilization.

### 3.2. Effect of Inoculum Ratio and Speed Agitation on Gluconic Acid Production in Synthetic Medium

**Figure 2:**
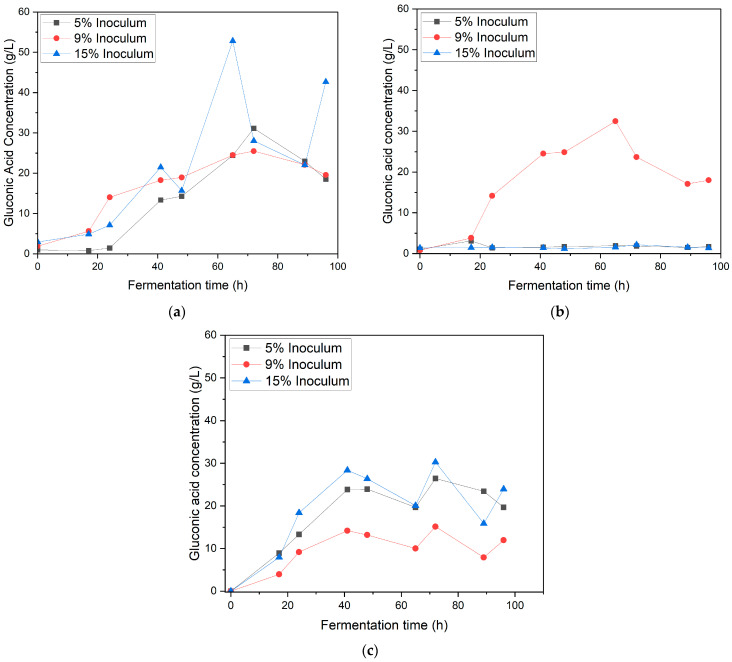
Gluconic acid concentration in synthetic medium at different inoculum ratios (5%, 9%, and 15%) under varying agitation speeds: (**a**) 150 rpm, (**b**) 190 rpm, and (**c**) 220 rpm.

Figure 2 shows the effect of varying the inoculum ratio (5%, 9%, and 15% *v*/*v*) and agitation speed (150, 190, and 220 rpm) on the concentration of GA during fermentation in the synthetic medium. At 150 rpm (Figure 2a), the highest GA concentration was achieved with the 15% inoculum, reaching 52.82 ± 12.88 g/L at 65 h. The 5% and 9% inoculums yielded 31.14 ± 3.38 g/L and 25.48 ± 7.59 g/L, respectively. These findings suggest that higher inoculum ratios enhance gluconic acid production, likely because of increased microbial density, facilitating more efficient substrate utilization [18]. Agitation at 150 rpm appeared to provide optimal oxygen transfer while minimizing shear stress, creating a favorable environment for microbial activity [19].

At 190 rpm (Figure 2b), the GA concentration decreased compared with that at 150 rpm. The 9% inoculum exhibited the highest GA concentration of 32.47 ± 2.32 g/L. The slight improvement in the performance of the 5% inoculum suggests that increased agitation enhances oxygen availability but may also introduce mechanical stress, which is particularly detrimental to larger inoculum sizes [18]. These results highlight the delicate balance between oxygen transfer and agitation-induced stress during fermentation.

GA production declined significantly at the highest agitation speed of 220 rpm (Figure 2c) for all the inoculum ratios. The 15% inoculum achieved a maximum GA concentration of only 30.3 ± 0.16 g/L, while the 5% and 9% inoculums yielded 26.45 ± 5.82 g/L and 15.15 ± 0.30 g/L, respectively. Excessive agitation likely causes increased shear stress and disrupts microbial metabolism, leading to reduced gluconic acid production [6]. This indicated that high agitation speeds were detrimental to gluconic acid fermentation by *G. oxydans*.

**Figure 3:**
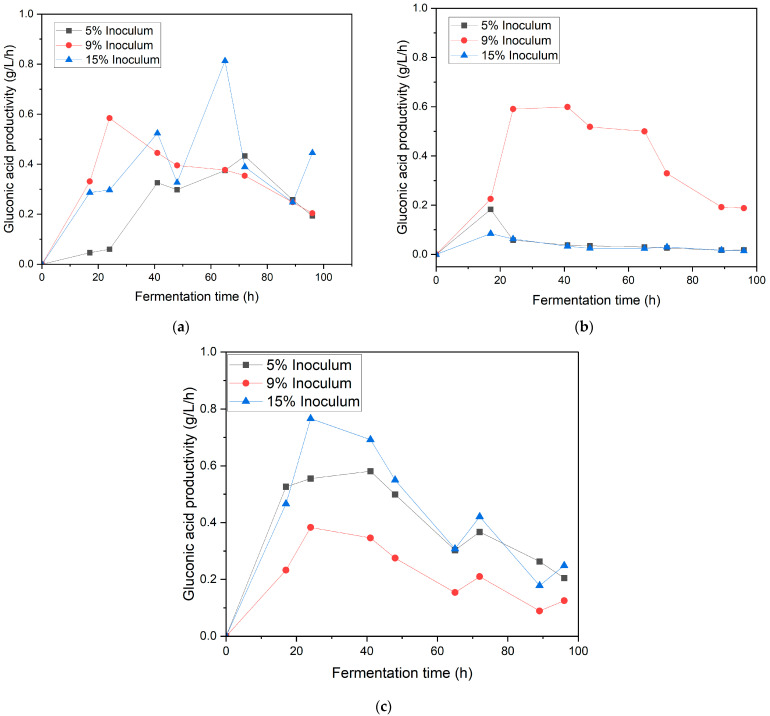
Gluconic acid productivity in the synthetic medium at different inoculum ratios (5%, 9%, and 15%) under varying agitation speeds: (**a**) 150 rpm, (**b**) 190 rpm, and (**c**) 220 rpm.

Figure 3 illustrates the production of GA under identical conditions, with variations in the inoculum ratios (5%, 10%, and 15% *v*/*v*) and agitation speeds (150, 190, and 220 rpm). At 150 rpm (Figure 3a), the 15% inoculum exhibited the highest peak productivity of approximately 0.81 ± 0.19 g/L/h, demonstrating its efficiency in converting substrate into gluconic acid. Productivity decreased with increasing agitation speeds, reaching approximately 0.59 g/L/h at 190 rpm (Figure 3b) and 0.55 g/L/h at 220 rpm (Figure 3c). These results further reinforce that moderate agitation and a higher inoculum ratio provide the most favorable conditions for maximizing gluconic acid production and productivity, effectively balancing oxygen transfer with minimal mechanical stress.

### 3.3. Effect of Inoculum Ratio and Speed Agitation on Xylonic Acid Production in Synthetic Medium

**Figure 4:**
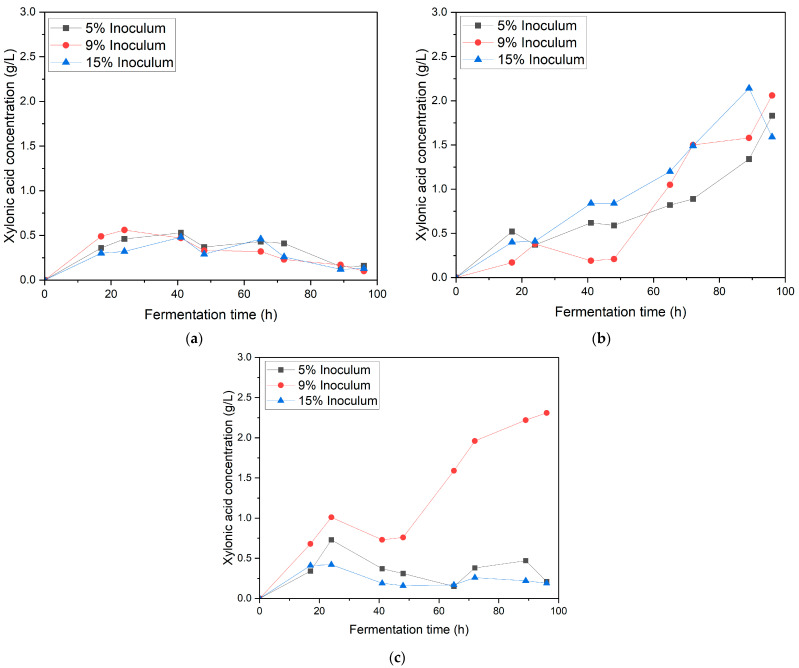
Xylonic acid production in synthetic medium at different inoculum ratios (5%, 9%, and 15%) under varying agitation speeds: (**a**) 150 rpm, (**b**) 190 rpm, and (**c**) 220 rpm.

Figure 4 depicts the effect of varying inoculum ratios (5%, 9%, and 15%) and agitation speeds (150, 190, and 220 rpm) on xylonic acid production in a synthetic medium during a 100 h fermentation period. At the lowest agitation speed of 150 rpm (Figure 4a), xylonic acid concentrations remained consistently low, ranging between 0.12 ± 0.06 and 0.56 ± 0.13 g/L across all inoculum ratios. This suggests that a low agitation speed of 150 rpm can hinder xylonic acid production by lowering the dissolved oxygen levels in the broth. Reduced oxygen availability impairs the catalytic efficiency of bacteria, ultimately resulting in diminished production [20].

Xylonic acid production showed a marked increase at an agitation speed of 190 rpm (Figure 4b), highlighting the benefits of moderate agitation in this process. Among the tested inoculum ratios, the 15% inoculum achieved the highest concentration, reaching approximately 2.0 ± 1.12 g/L at 90 h, followed closely by the 9% inoculum (1.8 ± 1.10 g/L and the 5% inoculum (1.5 ± 1.1 g/L). This increase can be attributed to improved oxygen transfer and distribution at 190 rpm, which ensures sufficient dissolved oxygen levels to support the aerobic metabolic pathways essential for xylonic acid synthesis. The enhanced mixing at this agitation speed also promotes uniform nutrient availability, reducing substrate limitations and fostering microbial growth and enzymatic activity [17].

**Figure 5:**
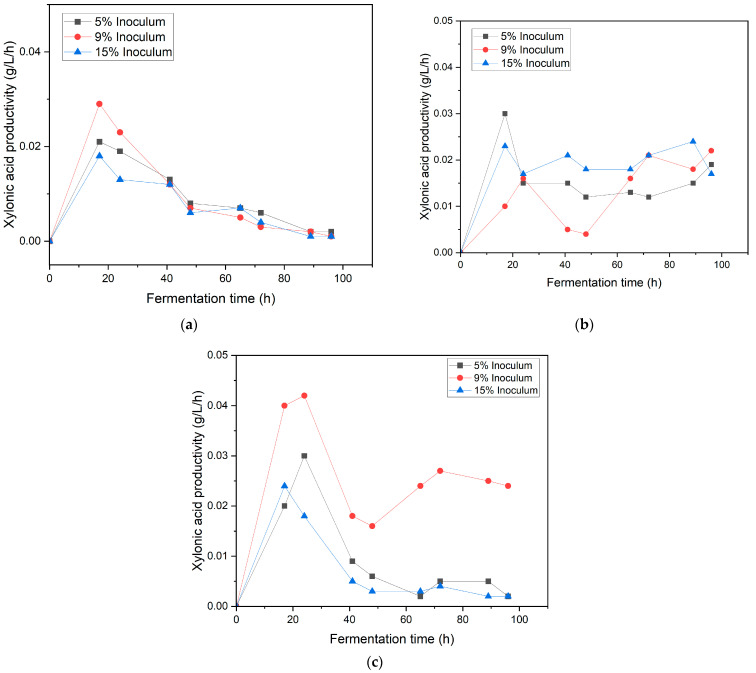
Xylonic acid productivity in the synthetic medium at different inoculum ratios (5%, 9%, and 15%) under varying agitation speeds: (**a**) 150 rpm, (**b**) 190 rpm, and (**c**) 220 rpm.

At the highest agitation speed of 220 rpm (Figure 4c), xylonic acid production reached its peak, with the 9% inoculum ratio achieving the highest concentration of approximately 2.31 ± 1.43 g/L. The 5% inoculum ratio yielded 0.7 ± 0.01 g/L, while the 15% inoculum ratio produced only approximately 0.6 ± 0.02 g/L. Xylonic acid productivity followed the same trend as that of xylonic acid production (Figure 5). This pattern suggests that while increased agitation improves oxygen transfer and nutrient distribution [17], excessively high inoculum ratios may cause metabolic stress or competition, reducing overall efficiency [12]. These findings highlight the importance of balancing the inoculum ratio and agitation speed to optimize microbial productivity during xylonic acid fermentation.

Figure 5 illustrates the impact of different inoculum ratios (5%, 9%, and 15%) and agitation speeds (150, 190, and 220 rpm) on xylonic acid productivity in a synthetic medium. At 150 rpm (Figure 5a), the 9% inoculum achieved the highest peak productivity of approximately 0.033 ± 0.003 g/L/h at 20 h, followed by the 5% inoculum with a peak of 0.02 ± 0.001 g/L/h and 15% inoculum at 0.01 ± 0.001 g/L/h. However, both ratios showed a decline in productivity afterward, suggesting nutrient or oxygen depletion at lower agitation rates [20,21]. At 190 rpm (Figure 5b), more stable productivity is observed for all inoculum levels, with 15% inoculum maintaining an average productivity of 0.02–0.03 g/L/h across the fermentation period, highlighting the benefit of improved oxygen transfer at moderate agitation. At 220 rpm (Figure 5c), the 9% inoculum again achieves the highest peak productivity, reaching approximately 0.04 g/L/h by 20 h, but the 15% inoculum experiences a decline after 40 h, likely due to shear stress caused by excessive agitation [17]. These findings suggest that a 9% inoculum performs optimally at higher agitation speeds (190–220 rpm), whereas a 15% inoculum benefits from moderate conditions but is sensitive to prolonged exposure to high agitation. These data emphasize the importance of balancing inoculum concentration and agitation to optimize xylonic acid production.

### 3.4. Effect of Inoculum and Speed Agitation on the Growth of Gluconobacter oxydans and the Production of Gluconic Acid and Xylonic Acid in Oil Palm Fronds Hydrolysate

**Figure 6:**
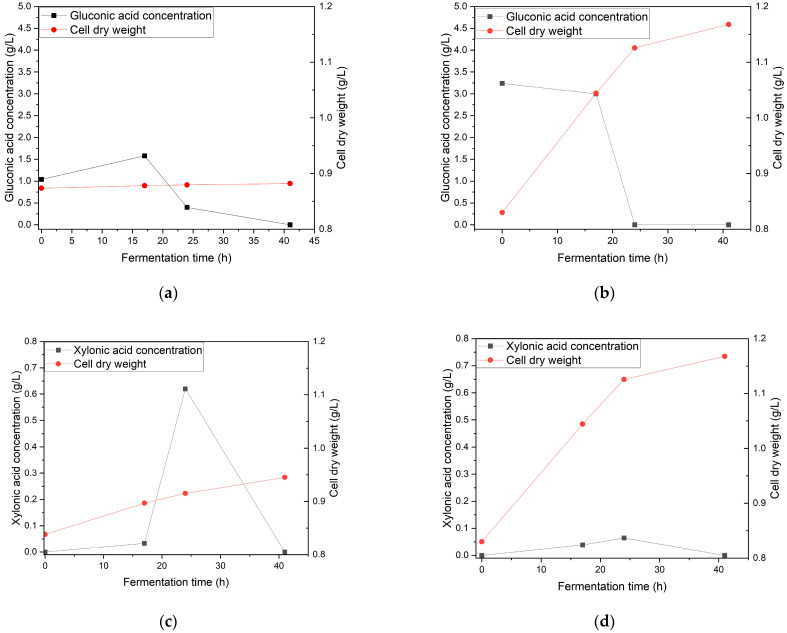
The growth of *G. oxydans* and the production of gluconic acid (**a**,**b**) and xylonic acid (**c**,**d**) in hydrolysate medium with variations 1 and 2.

The effects of inoculum concentration and agitation speed on the growth of *G. oxydans* and the production of gluconic acid and xylonic acid in the hydrolysate were investigated using two variations: (1) a 5% *v*/*v* inoculum ratio with 190 rpm agitation, and (2) a 15% *v*/*v* inoculum ratio with 220 rpm agitation. Based on Figure 6, the results revealed interesting patterns in the growth of *G. oxydans* and gluconic acid production across the two variations. In variation 1, the gluconic acid concentration starts at 1.0 ± 0.01 g/L and rises to 1.6 ± 0.01 g/L at 20 h before sharply declining to near 0 g/L by 40 h. This pattern can be attributed to the bacteria’s initial adaptation phase, during which they efficiently converted glucose to gluconic acid, followed by a depletion phase when glucose was limited [22]. Furthermore, the reduction in gluconic acid concentration was attributed to its conversion to 2-ketogluconic acid. Additionally, the gluconic acid produced undergoes oxidation and decomposition [23]. Cell growth in this variation increased steadily from 0.23 to 1.6 ± 0.01 g/L, indicating that a lower inoculum ratio provided favorable conditions for cellular metabolism and consistent nutrient utilization [16].

In contrast, variation 2 began with a higher initial gluconic acid concentration of 3.24 ± 0.66 g/L, which may have caused substrate inhibition, reducing the efficiency of cell growth despite the abundance of substrate. This phenomenon, in which excessive substrate levels overwhelm cellular transport mechanisms or create unfavorable osmotic conditions, results in lower cell growth than in variation 1 [24,25]. The influence of agitation speed on fermentation dynamics was also evident. Variation 1 demonstrated a more consistent cell growth trajectory, suggesting that an agitation speed of 190 rpm provided optimal oxygen transfer and substrate mixing for *G. oxydans*, a strictly aerobic bacterium. However, the sharp decline in gluconic acid concentration after 20 h in both variations and continued cell growth in variation 1 suggests a metabolic shift. Under sufficient oxygen conditions maintained by proper agitation, *G. oxydans* can metabolize gluconic acid as a secondary carbon source when glucose is scarce. In variation 2, reduced cell growth despite higher initial substrate levels indicated potential oxygen limitations caused by suboptimal agitation [17]. This emphasizes the critical role of agitation in maintaining adequate oxygen levels and a homogeneous substrate distribution throughout the fermentation medium.

As shown in Figure 6, the effect of inoculum on *G. oxydans* growth and xylonic acid production showed distinct patterns in both variations in the hydrolysate medium. In variation 1, the cell growth demonstrates a steady increase from approximately 0.09 to 0.72 ± 0.01 g/L over the 40 h fermentation period, while xylonic acid production reaches a peak of about 0.62 ± 0.04 g/L at 20 h before declining to near zero by 40 h. This pattern suggests that the initial inoculum concentration supported efficient cellular metabolism for growth [26], but the xylonic acid production was limited by substrate availability or product inhibition [27,28]. In variation 2, despite showing similar cell growth trends with variation 1, the maximum xylonic acid concentration is significantly lower (0.06 ± 0.03 g/L), indicating that higher initial cell density might have created competition for available nutrients or oxygen, resulting in reduced acid production efficiency [29]. This phenomenon can be attributed to the metabolic preference of *G. oxydans*, where cell maintenance and growth may prioritize acid production under certain conditions [30].

Agitation speed significantly influences fermentation dynamics, which is particularly evident in the differences between variations 1 and 2. The continuous cell growth in both variations suggests that the agitation speeds maintained sufficient oxygen transfer for cellular respiration [31]. However, the sharp decline in xylonic acid concentration after 20 h and the continued cell growth indicated a metabolic shift in the bacterial population. This shift likely occurs because *G. oxydans*, an obligate aerobe, requires optimal oxygen transfer for growth and acid production [32,33]. The lower xylonic acid production in variation 2 could be due to suboptimal mixing conditions, which might have created microenvironments with varying oxygen availability, affecting the bacteria’s oxidative capacity [30]. Furthermore, the relationship between cell growth and acid production suggests that *G. oxydans* preferentially utilizes the produced xylonic acid as a secondary carbon source under oxygen-sufficient conditions when the primary substrate becomes limiting, explaining the acid concentration decline while cell growth continues [30].

## 4. Conclusions

This study successfully established a sustainable bioprocess for converting by-products of the palm oil industry into valuable organic acids through microbial fermentation. Significant yields of GA and XA were obtained by optimizing key fermentation parameters, such as agitation speed, inoculum ratio, and media composition. In the synthetic medium, GA reached a maximum concentration of 52.82 ± 12.88 g/L at 65 h, while XA concentration achieved 2.31 ± 1.43 g/L at 96 h. In the hydrolysate medium, GA concentration reached 3.24 ± 0.66 g/L at the start of fermentation, and XA concentration of 0.62 ± 0.04 g/L was achieved at 24 h. These results highlight the robust bioconversion capabilities of *G. oxydans*, offering a scalable and environmentally friendly approach for the production of organic acids from agricultural waste.

## Data Availability

All data and materials are available upon request to the corresponding author. The data are not publicly available due to [privacy and confidentiality restrictions].
